# Number of Axillary Lymph Node Metastases Determined by Preoperative Ultrasound is Related to Prognosis in Patients with Breast Cancer

**DOI:** 10.3390/cancers2010020

**Published:** 2010-02-04

**Authors:** Yuko Kijima, Heiji Yoshinaka, Munetsugu Hirata, Tadao Mizoguchi, Sumiya Ishigami, Akihiro Nakajo, Hideo Arima, Shinichi Ueno, Shoji Natsugoe

**Affiliations:** Department of Surgical Oncology, Breast and Endocrine Surgery, Kagoshima University Graduate School of Medical and Dental Sciences 8-35-1, Sakuragaoka, Kagoshima 890-8520, Japan; E-Mails: heiji@m3.kufm.kagoshima-u.ac.jp (H.Y.); springman1021@yahoo.co.jp (M.H.); turboh-9@m.kufm.kagoshima-u.ac.jp (T.M.); igeka@m2.kufm.kagoshima-u.ac.jp (S.I.); anakajo@m.kufm.kagoshima-u.ac.jp (A.N.); h-arima@m3.kufm.kagoshima-u.ac.jp (H.A.); ueno1@m.kufm.kagoshima-u.ac.jp (S.U.); natsugoe@m2.kufm.kagoshima-u.ac.jp (S.N.)

**Keywords:** breast cancer, ultrasound, lymph node metastases, sentinel lymph node, prognosis, neoadjuvant therapy

## Abstract

Objective: To analyze the impact on prognosis of the number of axillary lymph node metastases (LNM) detected by ultrasound (US) in patients with breast cancer. Methods: One-to-one comparison of LNM was performed between the ultrasound and histologic diagnosis in 380 patients. Results: The accuracy of preoperative ultrasound diagnosis was 79.7%. According to the subdivision of number of LNM (0, 1–3, 4–9, 10+), the accuracy rates associated with LNM were 82%, 49%, 34%, and 86%, respectively. The disease-free-survival curves according to the number of LNM were similar in them. Conclusion: Preoperative ultrasound can determine axillary involvement and may be useful for predicting prognosis.

## 1. Introduction

Recently, the sentinel node concept has become acceptable as the standard strategy in the staging of the axilla in early stage breast cancer [1,2]. It has become more important to evaluate the presence or absence of lymph node metastases prior to surgery when planning the treatment strategy in breast cancer. 

The presence of lymph node metastasis, in the absence of distant recurrence, is the single most important prognostic factor in breast cancer [3] and postoperative adjuvant therapy should be selected according to the number of metastastic lymph nodes, tumor size, histological grade, vessel involvement, patient’s age, HER2 status and hormone receptor status [4]. The TNM classification of the International Union Against Cancer has incorporated the number of lymph node metastasis to breast cancer. According to the TMN classification, the number of regional nodal metastasis in pN1, pN2 and pN3 was 1–3, 4–9 and 10 or more, respectively [5]. The presence or absence of lymph node metastasis is not always necessary and it may be useful in neoadjuvant therapy or determination of need for postmastectomy radiation and reconstruction consideration. When performing the reduction of lymphadenectomy, even in sentinel lymph node biopsy, or planning induction chemotherapy, preoperative diagnosis of lymph node metastasis is also essential [6] .

In general preoperative assessment is not done and CT and MRI may suggest metastastic disease, however it is biopsy, fine needle aspiration cytology, core needle biopsy, sentinel lymph node biopsy that proves metastatic disease. The present study evaluated the accuracy and limitations of US in the preoperative diagnosis of lymph node metastases and especially in the impact of the number of lymph node metastases on the prognosis of patients with breast cancer. 

## 2. Results and Discussion

### 2.1. Lymph Node Metastasis

US predicted lymph node metastasis in 34.7% (132/380) of patients, whereas lymph node metastasis was confirmed by following examination of dissected lymph nodes specimen in 37.1% (141/380). US correctly diagnosed the presence of lymph node metastasis in 98 of 141 patients (69.5%) with histologically proven nodal involvement. The sensitivity and specificity of axillary lymph node metastases by preoperative diagnosis and histologic diagnosis was 69.5% (98/141) and 85.8% (205/239), respectively. Accordingly, the accuracy rate of US diagnosis was 79.7%(303/380, Table 1). 

**Table 1:** Diagnostic accuracy of ultrasound in detecting axillary lymph nodes with metastases.

US diagnosis	Histological Diagnosis		Total
	Positive Nodes	Negative Nodes	
Positive	98	34	132
Negative	43	205	248
Total	141	239	380

Sensitivity: 98/141 = 69.5%; Specificity: 205/239=85.8%; Accuracy: 303/380 = 79.7%.

**Figure 1:**
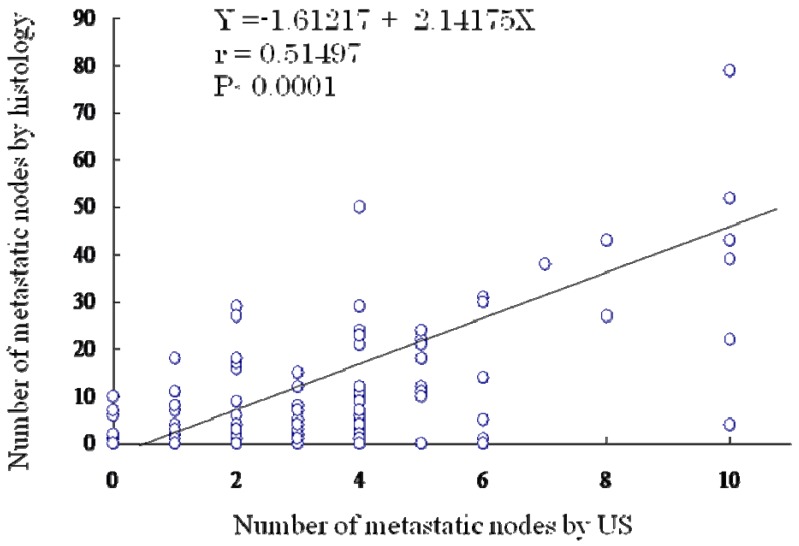
.Correlation of the number of lymph node metastases between ultrasound diagnosis and histologic diagnosis. A significant difference using multiple logistic analysis was found (P < 0.001; correlation coefficient [r] = 0.51497).

The total number of metastatic nodes identified by histology and by US was 8.7 and 3.1, respectively. Figure 1 shows the relationship of the number of lymph node metastases between preoperative diagnosis by US and histologic diagnosis. As a result of multiple logistic analysis, the correlation between them was significant (P < 0.001). 

### 2.2. Subdivision of the Number of Metastatic Nodes

The number of metastatic nodes was classified into subdivision of zero, one to three, four to nine, and ten or more (Table 2).

**Table 2:** Comparison between preoperative and histologic diagnosis according to the number of lymph node metastases.

		No. of Metastatic Nodes on Histological Diagnosis				Total No. of axilla
		0	1–3	4–9	10+	
No. of Metastatic Nodes on US Diagnosis	0	205	33	7	3	28
	1–3	28	38	6	5	77
	4–9	5	3	14	19	41
	10+	1	0	1	12	14
	Total No. of axilla	239	74	28	39	380

Among 248 patients in whom lymph node metastasis was undetectable by US, 205 (82.7%) were diagnosed as being free of nodal involvement by histology. Although 49.4% (38/77) of the patients who had one to three lymph node metastases were correctly diagnosed by US, 28 patients (36.4%) were underestimated. The accuracy rate of ultrasound diagnosis was 34.1% among patients with four to nine involved nodes. Histology confirmed 12 of 14 patients (85.7%) with ten or more involved nodes diagnosed by US.

### 2.3. Impact of Prognosis

The disease-survival was analyzed according to the number of lymph node metastases determined by US. The 5-year disease-free survival rate of patients with zero, one to three, four to nine, and ten or more lymph node metastases diagnosed by US were 93.8%, 76.6%, 57.7 %, and 36.3%, respectively. The significant difference was found between the patients without lymph node metastasis and those with one to three lymph node metastases (Figure 5). 

**Figure 2:**
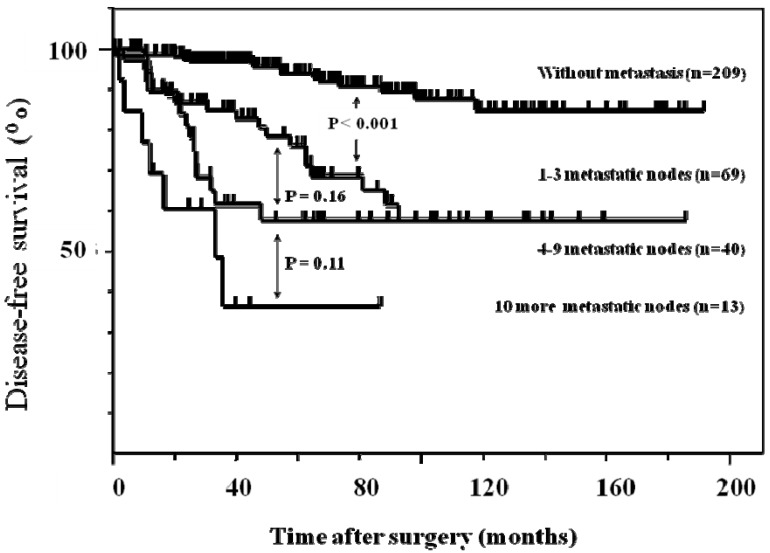
Five-year disease-free survival curves according to the number of lymph node metastases by ultrasonography.

While the 5-year disease-free survival rate according to histologic diagnosis was 95.1% in patients without nodal involvement, 82.8% in those with one to three metastases, 52.1% in those with four to nine metastases, and 45.0% in those with ten or more metastases (Figure 6). There was a significant difference among each group except for the group between the patients with 4–7 metastases and those with 10 or more metastases. 

**Figure 3:**
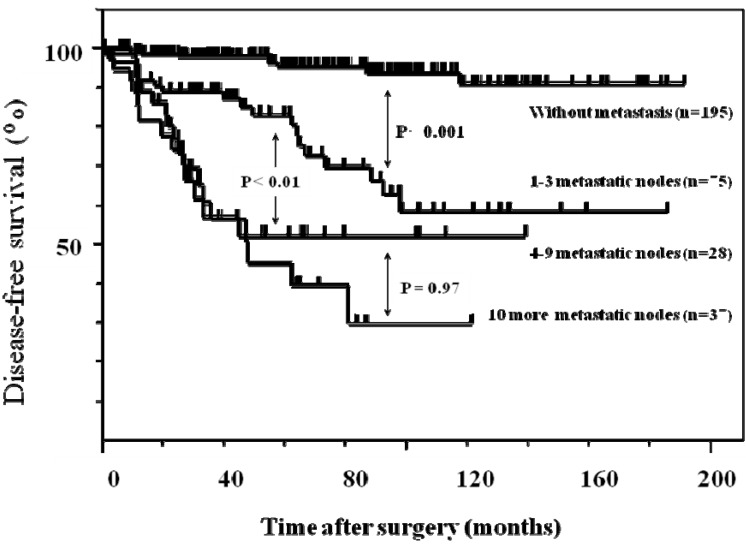
Five-year disease-free survival curves according to the number of lymph node metastases by histologic examination.

### 2.4. Discussion

Management and staging of breast cancer according to axillary nodal status has been the subject of intense debate and controversy [10,11,12,13,14,15]. The study of NSABP B-04, which randomized patients with clinically uninvolved axillary nodes to radical mastectomy, total mastectomy plus radiotherapy, or total mastectomy alone, demonstrated that axillary treatment with either dissection or regional radiotherapy reduced axillary recurrence rates from 18.6% to 1%–2%. However, there was no benefit to axillary treatment in terms of distant disease-free survival [16].

When diagnosing lymph node metastasis by ultrasound, it is important to examine not only the size of nodes but also the morphologic findings. In our study, ultrasonographic images of lymph nodes were classified on the basis of boundary and internal echoes [7,8,9]. We diagnosed a patient as having positive lymph nodes when detected lymph nodes met the criteria of both boundary and internal echoes. Preoperative diagnosis of lymph node metastasis is essential for determining the treatment strategy of breast cancer. In recent years, sentinel node biopsy has been generally acceptable, as an attempt to minimize overtreatment of healthy axilla [1,2,17]. One of the problems of sentinel node biopsy is the presence of false negative results. It has been reported that 1%–15% of patients with negative sentinel node biopsy had nodal metastasis in the same region [18], and the false negative rate of sentinel node biopsy has improved over time and is probably under 5% now in most experienced group [19]. When we analyzed relationships between metastatic area and radioisotope uptake in sentinel nodes of esophageal and gastric cancer patients, radioisotope uptake was not detectable in some lymph nodes with > 60% metastatic area. However, the false negative lymph nodes are not always probably the massive tumor involved ones, as one would hope they would be sampled intraoperatively and then go to axillary node dissection with grossy abnormal nodes. Thus, clinical diagnosis of lymph node metastasis, especially by US diagnosis, is essential before sentinel node navigation surgery [20]. 

An accurate imaging diagnosis of the axillary nodal metastasis is important for pretreatment staging of patients enrolled in neoadjuvant protocols [21], since survival curves were influenced by the number of metastastic axillary lymph nodes [13,22,23,24]. Valstos *et al*. reported that nearly half of patients for whom both physical and ultrasonography examinations of the axilla were changed as positive to negative after induction chemotherapy showed histologic evidence of nodal metastases. They also concluded that patients with positive preoperative findings on US examination should continue to receive axillary lymph node dissection in order to do sufficient local control [25]. In the present study, since the accuracy rate was 81.3%, the diagnosis of US is useful for selection of patients with induction chemotherapy. 

A close relationship between the number of lymph node metastases and the clinical outcome of patients with breast carcinoma has been reported. Involvement of more than 3 nodes, as indicated in the TNM staging system, is the crucial cut-off point for the prognostic categorization of patients [26]. However, the cut-off number of nodal metastasis is controversial in breast cancer. For example, Smith *et al*. [22] identified five lymph node metastases, while Mittra *et al*. [3] identified seven as the most significant cut-off point for survival. Fisher and the NSABP group indicated a prognostic difference between patients with 4–6 involved nodes and those with more than 12 [3]. Cascinelli *et al*. found that prognosis was poorer in two or more nodal involvement than in single involvement in patients older than 40 years [24]. 

However, most reports were based on histologic examination of lymph node metastases of lymph node metastases after surgery. In the present study, we prospectively evaluated the number of lymph node metastases by US before surgery and analyzed them at a one-to-one correspondence between preoperative and histologic diagnosis. The correlation between the number of lymph node metastases diagnosed before surgery and by histology was significant. We divided the number of involved nodes into groups of zero, one to three, four to nine, ten or more according to the TNM classification. Among patients without metastasis, the accuracy rate by US was high. The positive relationship between the number of preoperative diagnosis using ultrasonography and histological status might suggest us not only an estimated true number of metastastic lymph nodes but also the prognoses of patients while we could not get distinct data of metastastic status after neoadjuvant systemic therapy. 

On the other hand, false negative rate in patients with 1–3, 4–9 and 10 or more nodal metastases was 45.3%, 25.0% and 7.7%, respectively. As the number of metastastic lymph nodes was larger, the false negative rate of preoperative US diagnosis was lower. On the other hand, the rate of underestimated diagnosis for lymph node metastasis was high, as the number of metastatic nodes increased. The underestimation was caused by the tiny metastatic foci in the lymph node and the metastasis of small size nodes. Thus, we must keep in mind that the actual number of histological metastasis is larger than that detected by preoperative US diagnosis, especially in the patients of many metastases. 

In this series, the 5-year disease-free survival rates of patients were significantly different based on the four subdivisions of preoperative US diagnosis, except for the difference in patients between with 1–3 and with 4–9 metastases. The disease-free survival curves obtained after diagnosis by ultrasound were similar to those obtained by histologic diagnosis. These results suggested that the prognosis of patients with breast carcinoma can be predicted by the number of lymph node metastases based on preoperative US diagnosis. 

## 3. Experimental Section

### 3.1. Patients and Methods

Between January 1992 and March 2007, 386 consecutive Japanese patients with breast carcinoma were admitted to the Department of Breast and Endocrine Surgery at Kagoshima University Hospital, Japan. Informed consent was obtained from all the patients preoperatively. The design of the study has been approved retrospectively by the ethical committee of the Kagoshima University Hospital. Preoperative US examination was performed in all patients by one experienced surgeon (H.Y.). We did receive permission to use these US images. Of these, 20 patients with metachronous bilateral breast cancer and three patients with synchronous bilateral breast cancer were included in this study. We included those three patients into this study by counting independent axilla but count each case when we examine the prognostic results. Four patients with preoperative chemotherapy and five patients without lymphadenectomy were excluded. Thus, a total of 377 patients (380 axillas) were retrospectively studied. Three hundred thirty patients who underwent axillary lymphadenectomy with or without infraclavicular or supraculavicular or parasternal lymphadenectomy and a total of 50 patients who underwent sentinel lymph node biopsy without axillary lymph adenectomy were included in this study**.** Three male patients and 377 female patients ranged in age from 23 to 90 years (mean 57.4). Based on TNM classifications [5], Tis tumors were found in 26 patients, T1 in 147, T2 in 160, T3 in 19, and T4 in 28. Histologically, 24 tumors were ductal carcinoma *in situ*, 320 were invasive ductal carcinoma, two were lobular carcinoma *in situ*, and 11 were invasive lobular carcinoma, and the remaining 23 were special types of carcinoma. Out of them we enrolled only 320 patients with invasive ductal carcinoma and 11 with invasive lobular carcinoma when we examined the prognosis. Radical mastectomy was performed in 12 patients, modified standard mastectomy in 261, and breast conserving surgery in 107. The number of resected nodes in patients with axillary node alone and in patients with both axillary and infraclavicular lymph node ranged from 7 to 51 (mean 22.9) and 9 to 111 (mean 31.3), respectively. In patients with sentinel lymph node biopsy, the number of removed nodes ranged from 1 to 14 (mean 4.4). All of resected lymph nodes were fixed in 10% formalin and embedded in paraffin. A longitudinal cross-section through the hilus was made on all dissected nodes and carried out by HE staining. Larger carcinoma lesion than 0.2 mm in lymph node was considered as metastasis according to TNM classification [5]. Postoperative chemotherapy was added to 38 patients, endocrine therapy to 143 and both therapies to 107. Follow-up data after surgery were available for all patients and the average follow-up period was 77.5 months (range 2 to 210). The endopoint of this study was defined as a locoregional recurrence or distant metastases.

### 3.2. Ultrasonographic Examination and Criteria for Axillary node Evaluation

US examination was carried out using the radial mechanical scanners (Aloca SSD1200 or Aloca SSD1000 with a 10-MHz transducer) (Aloca, Co., Tokyo, Japan). The lymph nodes of axillary, infraclavicular, and supraculavicular regions were evaluated separately. 

As we reported, lymph nodes were classified into three types according to the boundary and internal echoes on the basis of the ultrasound appearance [7,8,9]. Type 1 lymph nodes had thin C-shaped boundaries with low echoic and regular cortex. 

**Figure 4:**
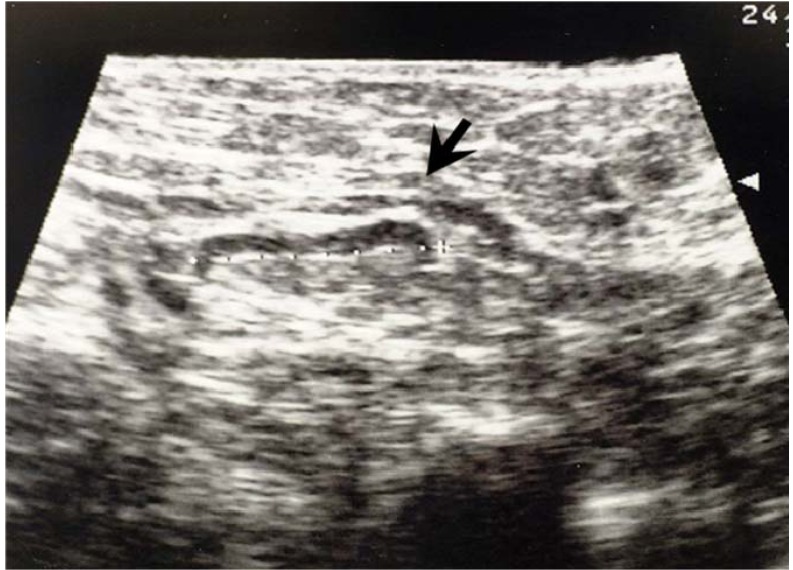
Ultrasonographic appearance of a normal lymph node (type 1). This lymph node (arrow) shows thin C-shaped low echoic cortex and regular cortex.

Type 2 lymph nodes had well-defined boundaries and weak and relatively sonolucent internal echoes. These lymph nodes often showed the disappearance of hilum fat hyperechogenicity and eccentric focal thickness and dent of cortex.

**Figure 5:**
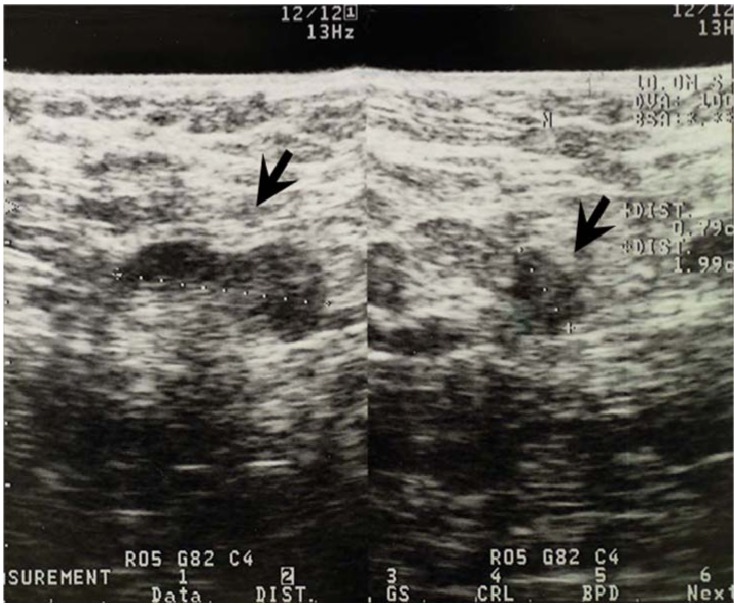
Ultrasonographic appearance of type 2 lymph node metastases. This lymph node (arrow) shows well-defined boundaries and weak internal echoes.

Type 3 lymph nodes had well-defined boundaries and often displayed notching and strong internal echoes.

**Figure 6:**
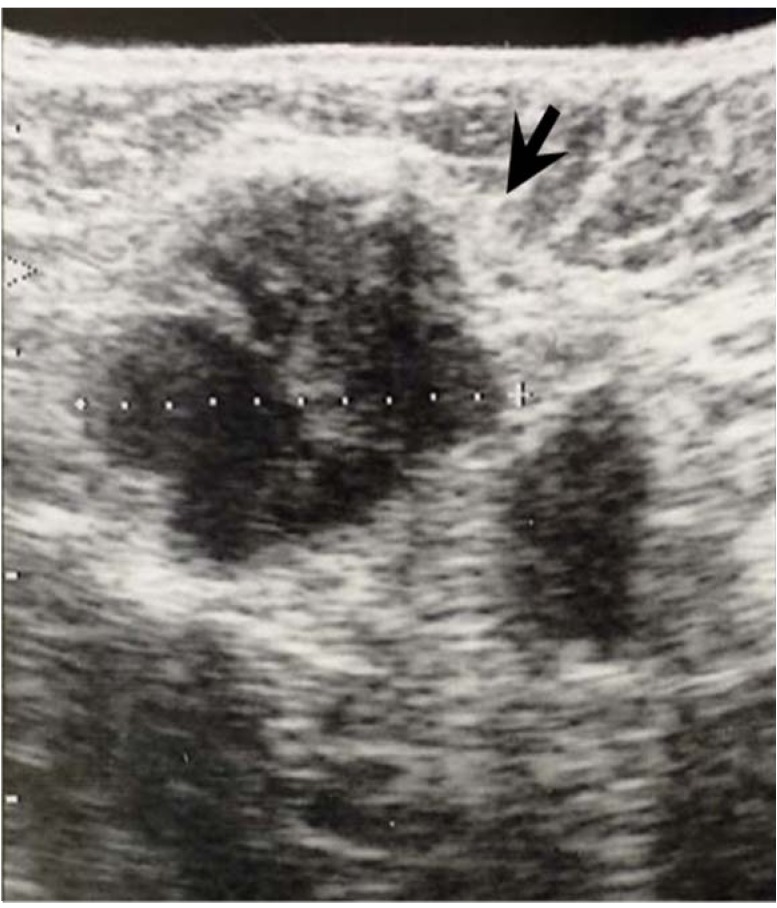
Ultrasonographic appearance of type 3 lymph node metastases. This lymph node (arrow) shows clearly defined boundaries but scattered large internal echoes accompanied by notching.

Type 1 lymph nodes were considered to be non-metastatic and type 2 or type 3 nodes were considered metastatic. All ultrasound films were evaluated by two independent observers (Y. K and H. Y). In cases in which the results of US image differed between the two observers, the films were evaluated by a third observer (Y.F). The accuracy and prognosis by US diagnosis was compared with histological diagnosis after the number of metastatic nodes was divided into 4 subclassification; 0, 1–3, 4–9 and 10 or more according to the TNM classification. 

### 3.3. Statistical Analysis

The data were evaluated using Stat Flex 4.2 software package (Artec Institute, Inc., Tokyo). In analyzing the data between preoperative US diagnosis and histological ones was statistically analyzed by the chi-square test and logistic regression analysis．Survival data were analyzed using the Kaplan-Meier survival model and are expressed as observed disease-free survival. The endopoint of this study was defined as a locoregional recurrence or distant metastases. The log-rank test was used to assess statistical differences between the groups. P < 0.05 was considered significant. Survival data was discussed only between the cases with invasive carcinoma: 11 cases with invasive lobular carcinoma and 320 ones with invasive ductal carcinoma.

## 4. Conclusions

In summary, there was a close relationship between preoperative US diagnosis and histologic diagnosis in the number of lymph node metastases. Therefore, US is useful when estimating the prognosis of patients with breast carcinoma. In the future, the preoperative number of lymph node metastases may be used in the staging system and the determination of neoadjuvant chemotherapy. We declare that there are no financial relationships or other interests with regard to this manuscript that might be construed as constituting a conflict of interest for any author.
